# Respiratory and Systemic Toxicity of Inhaled Artificial Asian Sand Dust in Pigs

**DOI:** 10.3390/life11010025

**Published:** 2021-01-04

**Authors:** Keon Kim, Seon-Deuk Kim, Tae-Hoon Shin, Chun-Sik Bae, Taeho Ahn, Sung-Shik Shin, Ha-Jung Kim, Chang-Min Lee, Guk-Hyun Suh

**Affiliations:** 1 Department of Veterinary Medicine, College of Veterinary Medicine and BK21 FOUR Program, Chonnam National University, Gwangju 61186, Korea; 206900@jnu.ac.kr (K.K.); clamc@naver.com (S.-D.K.); csbae210@jnu.ac.kr (C.-S.B.); thahn@jnu.ac.kr (T.A.); sungshik@jnu.ac.kr (S.-S.S.); kimhj614@jnu.ac.kr (H.-J.K.); 2 Translational Stem Cell Biology Branch, National Heart, Lung and Blood Institute, National Institutes of Health, Bethesda, MD 20892, USA; thshin1125@gmail.com

**Keywords:** Asian sand dust, domestic animal model, respiratory toxicity, systemic inflammation

## Abstract

Air pollution, particularly caused by Asian sand dust (ASD) and particulate matter (PM), has become one of the leading threats to public health. However, the majority of studies have primarily focused on epidemiological assessment, and in vivo toxicities of certain air pollutants have been poorly elucidated in medium/large-size laboratory animals. To investigate the impact of ASD in domestic animals, 16 Landrace pigs were exposed to an artificial ASD sandstorm for 6 h. All animals were divided in four cages, and a commercial yellow soil was used for generating artificial mineralogical particles. Blood samples were collected, and necropsies were performed before exposure and 6, 12, 24, and 72 h after exposure. Complete blood cell count and the levels of serum biochemical enzymes, blood gas, electrolytes, and a variety of inflammatory cytokines were evaluated. In addition, histopathological examination was conducted. Various test results proved acute lower airway disorders with systemic inflammation in pigs. To our knowledge, this study is the first to describe experimental research in domestic animals concerning the damage caused by artificial ASD exposure. The results of this study suggest that ASD has importance in terms of not only public health but also of ultimate economic losses in the pork industry.

## 1. Introduction

Air pollution has emerged as one of the leading threats to public health and quality of life [1]. Particularly, in the Asia-Pacific region including Korea and Japan, seasonal exposure to Asian sand dust (ASD) and/or airborne particulate matter (PM), originating from the East Asian deserts and mainland China, has been closely associated with environmental health concerns of people and animals living in this area [2,3,4]. ASD consists of diverse chemical and biological materials, such as sulfate, nitrate, polycyclic aromatic hydrocarbons, pollen, bacteria, and fungi [5,6], which can lead to incidence or exacerbation of symptoms of the respiratory or cardiovascular systems. Respiratory symptoms resulting from various inflammatory or allergic responses progressively worsen during the ASD peak period [7]. Moreover, numerous epidemiological studies have demonstrated a strong association between exposure to ASD and high risks of cardiovascular and respiratory events and mortality [3,8,9]. However, only a few investigations on in vivo toxicities of ASD in domestic animals are available to date.

Previous studies have demonstrated that airway exposure of experimental animals to ASD mineral particles, which are free from chemical and biological pollutants, induces pulmonary and systemic inflammation [10,11]. A similar ASD mineral particle exposure in tracheobronchial lymph nodes in dogs was reported [12]. In addition, one report suggested that ASD affects the respiratory system of domestic animals, such as sheep and goats, exposed to severe sandstorms in Mongolia [13]. Likewise, adverse effects of natural occurring ASD have already been reported on respiratory organs, with systemic inflammation in multiple animals.

ASD is a recent concern for the health of exposed inhabitants and animals [5]. Especially, it is important for domestic animals in terms of productivity. To the best of our knowledge, there have been few studies assessing the effects of ASD in domestic animals. Further, there was no report related to pigs, which are important domestic animals. Only epidemiological reports were published regarding the respiratory toxicity of domestic animals in the areas influenced by sand dusts [5]. This study aimed to investigate the adverse effects of ASD on respiratory organs and systemic inflammation in pigs subjected to artificial ASD inhalation.

## 2. Materials and Methods

### 2.1. Preparation of Artificial ASD

Based on the result of a full content analysis over several candidates, a commercial yellow soil of 5000 mesh (Yellow soil, HongikBio, Korea) was selected as a source of artificial ASD for this study because of its similarities to ASD with respect to concentration of water-soluble components and composition of total elements. The size distribution of the artificial ASD diameters ranged between 0.025 and 10 μm. The ASD particles were sterilized at 121 °C for 30 min and then stored in a freezer at −20 °C to prevent the growth of bacteria and fungi.

### 2.2. Animals

A total of 16 weaned Landrace pigs were included in this study. All pigs weighed approximately 15 kg and did not have any history of disease and health abnormalities. The animals were housed in groups under conventional conditions of 21–23 °C and 12-hour light/dark cycle in the animal facility of Chonnam National University, and water and food were supplied without restriction. All experiments conducted in this study were approved by and followed the instructions of the University of Institutional Animal Care and Use Committee (approval number: CNU IACUC-YB-2009-9, Chonnam National University, Gwangju, Republic of Korea).

### 2.3. Experimental Protocol

Four cages (1.2 m × 2.0 m) were compartmented in a polyethylene vinyl-coated chamber measuring 4.8 m × 2.8 m × 2.0 m (26.66 m^3^) in size after drug disinfection and formalin fumigation. All pigs were divided randomly into four groups. The four cages corresponding to the experimental groups, named as groups A, B, C, and D, were composed of four pigs each. Sixteen pigs were exposed to the artificial ASD in the form of an aerosol generated by the dust generator (TE740, Yamada, Japan) for 6 h at a rate of 250 g per minute.

Blood samples were drawn from the external jugular vein of each pig in groups A, B, C, and D at consecutive time points of 6, 12, 24, and 72 h after exposure and pre-inhalation to assess laboratory parameters and cytokine concentrations. At each time point of 6, 12, 24, and 72 h post-exposure, one pig from each of the four groups was randomly selected and euthanized by electric shock, and necropsies were conducted to evaluate the ASD-mediated histopathological lesions in the respiratory system.

### 2.4. Laboratory Blood Tests

To evaluate the overall pathological course in animals in response to ASD exposure, several hematological examinations were performed in the blood samples obtained at each time point. The automatic electrolyte and blood gas analyzer (Sysmex KX-21 autoanalyzer, TPA Medical Electronic Co., Kobe, Japan) was used to measure the concentration of venous carbon dioxide partial pressure (pCO2), sodium, potassium, lactate, and bicarbonate. The complete blood count (CBC) was performed using the automatic hematology analyzer for various species (HEMAVET 850, CDC, Oxford, CT, USA) to evaluate the circulating cellular components, such as total white blood cell (WBC) count, neutrophil, eosinophil, basophil, lymphocyte, monocyte, and red blood cell (RBC) parameters. A blood chemistry panel comprising evaluation of the values of total protein, albumin, blood urea nitrogen, creatinine, and other serum enzymes was performed by using an automatic serum chemistry analyzer (M-2300, Metrolab, Athens, Greece).

### 2.5. Cytokine Analysis

The concentrations of major pro-inflammatory cytokines, such as interleukin (IL)-4, IL-12, interferon gamma (IFN-γ), and tumor necrosis factor alpha (TNF-α), in the serum collected at; before; and 6, 12, 24, and 72 h after ASD exposure were measured using commercial enzyme-linked immunosorbent assay kits (Uscnlife Science & Technology CO., Wuhan, China).

### 2.6. Pathological Examination

During autopsies following electric shock, gross evaluation of the nasal cavity, trachea, and lung was initially conducted in each pig. Ten respiratory tissue samples were collected from dispersed locations in the trachea, bronchi, bronchioles, and alveoli, and these samples were fixed by immersion in 10% neutral-buffered formalin and embedded in paraffin after processing with alcohol and xylene. Serial sections of approximately 3-μm thickness were prepared and stained with hematoxylin and eosin.

Infiltration of artificial ASD particles in the lower respiratory tract, including the bronchus, bronchiole, and alveolus, was assessed over time via a microscopic examination in at least 14 different fields. A pathologist performed all histopathological evaluations to examine tissue integrity and evidence of inflammation in a blind manner.

### 2.7. Statistical Analysis

All data are shown as the mean ± standard deviation. Statistical significance was determined by using the Kruskal–Wallis test by ranks for multiple comparison. It extends the Mann–Whitney test, which is used for double screening for comparing only two groups. For all comparisons, *p* values less than 0.05 were considered statistically significant.

## 3. Results

### 3.1. Pigs Exposed to ASD Have a Predisposition to Systemic Inflammation and Organ Dysfunction

To assess systemic inflammation in response to artificial ASD inhalation, total cell counts were determined in the whole blood samples from pigs in each group at different time points including before and after exposure (6, 12, 24, and 72 h) (Table 1). Similar WBC count values were recorded before exposure to 24 h after the ASD exposure. Although there was no evidence of stochastic dominance statistically in the Kruskal–Wallis test, the mean WBC count at 72 h after the initial ASD exposure was elevated compared to that before ASD exposure. The number of neutrophils also increased at 72 h after exposure in comparison to that before exposure. Although the number of monocytes and eosinophils fluctuated initially after ASD exposure, an approximately two-fold increase in the monocyte and eosinophil counts was noted after 72 h compared to pre-exposure. While the increase in monocyte count was statistically significant, that of the eosinophil count was not. Moreover, the number of lymphocytes and basophils increased after ASD exposure, albeit no statistical significances were detected. This overall increasing tendency of all types of WBCs suggests variable types of systemic inflammation after exposure to the artificial ASD. However, the small number of animals used is a limitation for generalization. In contrast, the values related to RBCs and their function did not show any consistent results after exposure to ASD. However, the erythrocyte count showed an increase at initial exposure.

To examine the evidence of impairments or abnormalities in systemic organs, we then measured the levels of certain biochemical enzymes, electrolytes, and venous gases in blood samples obtained from different time points following artificial ASD exposure. The majority of serum biochemical enzymes did not display any significant changes, with the exception of albumin. The concentrations of albumin significantly decreased (*p* < 0.05) at 72 h after ASD exposure (1.91 ± 0.10 g/dL) compared to pre-exposure (3.43 ± 0.26 g/dL). Notable blood gas and electrolyte levels are summarized in Table 1. The concentration of pCO2 was increased gradually after ASD exposure in comparison to that before exposure, which suggests that the gas exchange system in the lungs had not worked appropriately. In addition, a mild increase in lactate level was noted at 72 h after the ASD exposure compared to that before exposure. With regard to blood electrolytes, while sodium level did not show any remarkable changes after ASD exposure, potassium level was increased at 72 h after ASD exposure compared to the concentration before exposure (*p* < 0.05). However, it should be kept in mind that the results of the Kruskal–Wallis test did not show stochastic dominance statistically (*p* = 0.08). Other significant changes were not detected in blood gas and electrolytes.

### 3.2. ASD Inhalation Provokes Systemic Hyperinflammatory Responses in Domestic Animals

**Figure 1:**
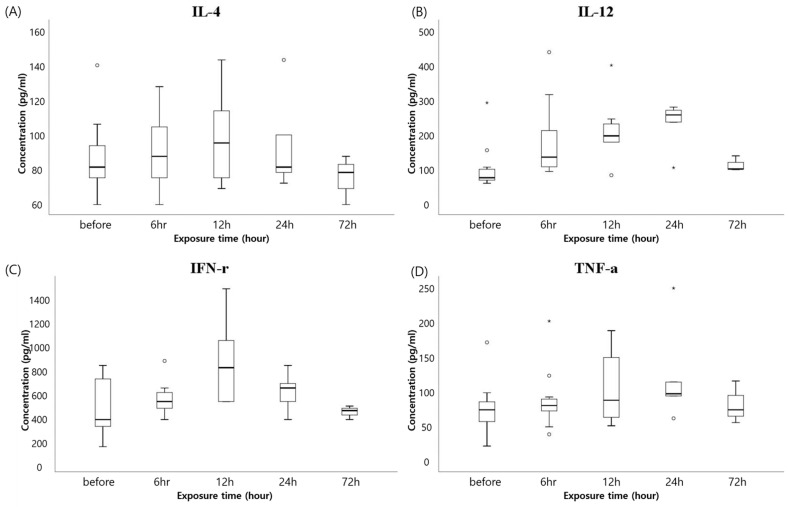
Concentration of inflammatory cytokines over time along exposure of artificial ASD in pigs: all concentration values depicted in these graphs were obtained by performing enzyme-linked immunosorbent assay analysis. All four graphs show the tendency of cytokine levels at 72 h after exposure, returning to the level before exposure or lower. Interleukin-4 and interferon gamma display similar patterns in concentration change, while interleukin-12 and tumor necrosis factor alpha have other inclinations. Each graph represents interleukin-4 (**A**), interleukin-12 (**B**), tumor necrosis factor alpha (**C**), and interferon gamma (**D**).

For more exact evaluation of systemic inflammation, we measured the serum concentration of major inflammatory cytokines at different times after the artificial ASD exposure in pigs. Compared to the samples drawn before ASD exposure, the levels of all measured inflammatory cytokines were increased following the ASD exposure and then recovered to values similar to the initial concentration after 72 h. IL-4 and IFN-γ exhibited similar patterns of reaching a peak around 12 h after exposure and then decreasing by the end of the follow-up period (Figure 1A,C). Meanwhile, the concentrations of IL-12 and TNF-α continued to increase around 24 h after exposure and thereafter decreased steeply (Figure 1B,D). Changes in all inflammatory cytokines were observed prior to an increase in WBC counts.

### 3.3. Experimental ASD Exposure Causes Toxicity in the Porcine Respiratory System

The degree of infiltration of ASD particles over time in the respiratory tract of pigs that were exposed to ASD was found (Table 2). As all 16 weaned pigs were young and had no history of exposure to ASD, the possibility of prior presence of particles in the respiratory tract was denied. ASD particles were observed in the entire respiratory tract, including the bronchus, bronchioles, and alveolus of the exposed pigs, and the highest percentage of infiltration was detected in pigs exposed at 6 h. In the case of pigs necropsying after exposure to ASD for more than 6 h, the infiltration of ASD particles gradually decreased. The ASD particles were most frequently detected in the bronchioles, while a few ASD particles were observed in the bronchi.

**Figure 2:**
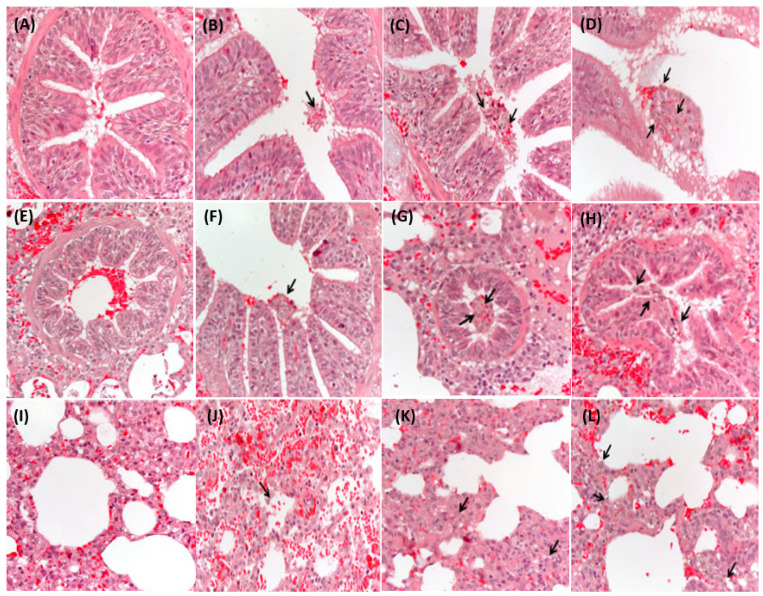
Histopathological images of lower the respiratory tract over time in pigs associated with experimental exposure of artificial ASD: the images show the bronchus at 6 h (**A**), 12 h (**B**), 24 h (**C**), and 72 h (**D**); the bronchiole at 6 h (**E**), 12 h (**F**), 24 h (**G**), and 72 h (**H**); and the alveolus at 6 h (**I**), 12 h (**J**), 24 h (**K**), and 72 h (**L**) after exposure. There were varied degrees of hemorrhagic evidence after ASD exposure in all images. Note the incline of increasing size in exogenous particles in all lower respiratory tract (arrows) since the exposure of artificial ASD in pigs. Any significant infiltration of leukocytes such as neutrophils was not detected. Each pig was chosen randomly for autopsy from groups A, B, or C at each time interval (hematoxylin and eosin stain; 400×).

The histopathological features in the respiratory tract displayed infiltration of ASD particles (Figure 2) in chronological order. The mucosal exudates were observed from the trachea to the distal part of bronchioles but were especially prominent in the bronchioles. The mucous membrane of the trachea was maintained with integrity continuously as the utmost position. In histopathological evaluation of the bronchus, most of the artificial ASD particles were found in the lumen space, whereas they were rarely attached to the cilia of the epithelium (Figure 2A–C). In the bronchioles, the ASD particles were detected with a mixture of exudates similar to that seen in the bronchi (Figure 2D–F). The ASD particles in the alveoli were attached to the alveolar epithelium rather than the lumen or were phagocyted by the alveolar epithelial cells (Figure 2G–I). Although there was no evidence of prominent inflammatory disorders in the respiratory tract, clinical respiratory symptoms, such as severe coughing, were detected in pigs after ASD exposure.

## 4. Discussion

This study describes acute respiratory toxicity and systemic inflammation induced in pigs by exposing them to the mineralogical components of artificial ASD. The possible mechanism for pathological changes is discussed by analyzing the histopathological examination results and hematological indices.

The number of neutrophils and monocytes, which are the hematological indicators of acute inflammation, showed an increase in levels at 72 h after ASD exposure. The tendency of increased total WBC count suggested that acute systemic inflammation was accelerated at 72 h after artificial ASD exposure. In addition, an increase in eosinophil count at 72 h after exposure indicated the possibility that the allergic reaction occurred simultaneously due to the ASD particles. A rapid increase in RBCs after ASD exposure is regarded as the body compensating for the reduction in oxygen delivery to the tissues. This compensation may be caused by the fine particles disturbing gas exchange in the alveoli. In contrast, hemoglobin concentration showed a decreasing pattern at initial ASD exposure. Previous studies have reported that iron availability could be limited in the acute phase of inflammation [14,15]. Limited iron availability could affect the entire mechanism of hematopoiesis. This might ultimately lead to a reduction in hemoglobin levels even though the level of increased RBCs was stabilized.

With the interpretation of CBC results, one significant change in serum chemistry could be expected: as the time interval increased, an accelerated phase of acute inflammation was noted. Albumin is known as a negative acute-phase protein produced in the process of inflammation [16]. A significant decrease in the albumin level at 72 h after ASD exposure could be affected by this mechanism. Blood gas analysis revealed an increase in pCO2 concentration compared to that before ASD exposure. An increased pCO2 concentration could be interpreted as a disturbance of gas exchange in the alveoli. In addition, an accelerating phase of inflammation at 72 h after ASD exposure could be explained by an increased potassium level. A notable increase in potassium level at 72 h after ASD exposure could be derived from cell destruction during the process of inflammation. However, additional research is needed to verify this assumption accurately in the future.

IL-4 is a cytokine that induces differentiation of naive T helper (Th) cells to Th2 cells [17]. TNF-α is required for IL-12-induced development of Th1 cells, and the Th1 phenotype has the capacity to secrete high levels of IFN-γ [18]. Considering an increase in the levels of these cytokines over time, it could be expected that both humoral and cell-mediated immunity are linked to exposure to artificial ASD. In addition, IL-4 is mainly responsible for the production of immunoglobulin (Ig) E, which is strongly associated with allergies, by eliciting Ig class switching in response to stimuli [19]. Moreover, the increase in eosinophil count at 72 h after exposure implies that an allergic reaction to the ASD particle occurred in this study.

As shown in Figure 1, IL-4 values reached the highest level at 12 h after ASD exposure. In contrast, other cytokines except IFN-γ did not attain a peak until around 24 h after ASD exposure. According to the previous studies, IL-4 decreased the productivity of inflammatory cells, such as Th1 cells and macrophages. Additionally, it can disturb the secretion of IFN-γ, TNF-α, and IL-12 derived from dendritic cells [20]. Because of the increased IL-4 levels, stagnation of IL-12 and TNF-α levels could be detected around 12 h after ASD exposure. However, at that time, stagnation in IFN-γ levels was not displayed exceptionally. This phenomenon could be explained by the function of IL-12. IL-12 reduces IL-4-mediated suppression of IFN-γ and stimulates the production of many inflammatory cytokines [21]. Consequently, IL-12 displayed a similar pattern of cytokine secretion over time, as shown in Figure 1. However, as an assumption is based on the only “cytokine value change”, further studies are needed to verify the origin of cytokines.

The microscopic examination revealed that all parts of the lower airway were extensively surrounded by ASD particles at 6 h after exposure and showed a diminishing tendency after then (Table 2). Considering that the histopathological evaluation showed exudates and the size of particle were gradually increased, particles may be congregated with one another over time. Although the portion of the airway where the particles could be detected was reduced, congregation suggested that the size of particles had been large enough to cause inflammation. A cluster of small congregated particles was able to irritate the entire lower airway and, consequently, cause catarrhal inflammation, as shown in Figure 2.

The bronchi have the widest diameter in the lower airway, and the size of the ASD particle is small enough to pass through the airway. Considering these facts, it is reasonable to think that only a few ASD particles could be detected in the bronchus. In contrast, these facts mean that the artificial particles could be detected mainly in the lumen of the bronchi. In the alveoli, particles were not found in the lumen significantly in spite of the small diameter. Instead, the ASD particles were deposited in the epithelial cells since the alveoli were positioned at the terminal site of the lower airway. Also, in the same context, phagocytosis of foreign materials noted in this area could be understood as the result.

Catarrhal inflammation was prominent especially in the area of the bronchioles. The smaller diameter of the bronchioles enabled artificial particles to be deposited easily rather than in the bronchi. However, although the alveoli had the smallest diameter, they did not show any evidence of catarrhal inflammation due to the absence of goblet cells in the alveoli. While a previous epidemiological report on domestic animals showed granulomatous inflammation [13], this study showed only catarrhal inflammation without any evidence of granulomatous inflammation. As necropsy was performed at the acute phase after exposure to ASD particles, it was expected that chronic damage by particles could not occur. As per the lung toxicity described in previous reports, this study suggests that ASD particles have the ability to induce both acute and chronic inflammation.

In the pork industry, viral diseases, such as porcine reproductive and respiratory syndrome, are known as an economically important factor not only limited in the Republic of Korea but also worldwide [22]. Furthermore, respiratory disorders in pigs are known to have multifactorial origins. Especially, the consequence of viral and bacterial infections is enhanced by poor environmental conditions [4]. In this study, the toxicity of artificial ASD itself was not prominent in destroying the structure of the respiratory system. However, it increased inflammation in the lower airway. The occurrence of inflammation in the airway may contribute to vulnerability of the structure against viral and bacterial infections. In conclusion, this study suggests that ASD not only could provoke inflammation but also can cause porcine respiratory disease complex. Therefore, it could be inferred that the importance of preventing exposure to ASD cannot be overemphasized in the porcine industry.

## 5. Conclusions

Airborne PM has an importance in public health and is known as the cause of respiratory and systemic disorders. Desert sand dusts are major components of airborne PM, of which the diameter is defined under 10 μm [2]. To our knowledge, there have been few studies that assessed the lung toxicity of ASD in vivo in a detailed manner. Although there were some experimental studies verifying the lung toxicity of ASD, all of these studies were performed in rodents [5,23]. Except for the abovementioned studies, there have been only epidemiological studies reporting the influence of ASD on the lungs of humans and other animals [10,13,24,25]. In this study, respiratory toxicity caused by ASD particles was evaluated deductively in domestic animals for the first time. Additionally, this study is novel in that it evaluated the changes after ASD exposure by both histopathological examination and blood analysis.

Recently, as global warming is becoming severe, desertification has been accelerated worldwide. Consequently, an increased exposure to PM including ASD is a crucial problem for both the veterinary and medical fields. Through this study, we verified that pigs could be damaged systemically and locally in the respiratory system. With regard to this environmental aggravation, this verification suggests requirements for precaution to prevent ASD exposure in pigs, ultimately leading to massive economic losses in the pork industry. Furthermore, the adverse effects of ASD exposure in humans could be verified indirectly by showing the toxicity of ASD in a porcine experimental model.

## Figures and Tables

**Table 1:** The results of selective blood works including cell count, blood gas, and electrolytes presenting significance over time along the exposure of Asian sand dust in pigs.

Blood Indices	Before Exposure of Artificial ASD	After Exposure of Artificial ASD			
		6 h	12 h	24 h	72 h
	N = 16	N = 12	N = 9	N = 6	N = 3
WBC (K/μL)	19.24 ± 6.70	22.25 ± 6.31	21.06 ± 4.28	19.14 ± 4.52	33.95 ± 11.41
NEU (K/μL)	6.70 ± 3.75	6.51 ± 2.23	6.87 ± 3.09	5.80 ± 1.87	11.36 ± 2.62
LYM (K/μL) ^†^	6.57 ± 3.26	9.35 ± 2.98	9.12 ± 2.28	8.72 ± 2.50	10.11 ± 3.76
MONO (K/μL) ^†^	2.01 ± 0.79	1.84 ± 0.72	2.31 ± 0.97	1.71 ± 0.78	4.63 ^a^ ± 2.22
EOS (K/μL)	3.78 ± 2.47	3.75 ± 2.45	2.71 ± 1.35	2.85 ± 1.20	7.04 ± 3.20
BASO (K/μL) ^†^	0.12 ± 0.10	0.12 ± 0.08	0.05 ± 0.04	0.06 ± 0.01	0.63 ± 0.51
RBC (M/μL)	6.17 ± 0.08	13.73 ± 3.87	11.38 ± 2.51	10.22 ± 1.71	13.22 ± 4.71
Hb (g/μL)	16.33 ± 4.69	14.35 ± 3.46	9.38 ± 3.25	11.02 ± 1.68	13.20 ± 2.76
MCV (fL/μL)	51.03 ± 5.27	55.59 ± 1.77	49.00 ± 2.69	49.53 ± 2.55	61.10 ± 1.31
ALB(g/dL) ^†^	3.43 ± 0.26	3.15 ± 0.52	3.66 ± 0.91	3.37 ± 0.91	1.91 ^b^ ± 0.10
pCO_2_ (mgHg) ^†^	59.67 ± 8.82	68.15 ± 11.37	70.78 ± 6.53	73.00 ± 5.22	79.00 ± 19.97
HCO_3_^−^ (mEq/L)	28.65 ± 5.35	31.17 ± 3.97	30.06 ± 4.07	31.42 ± 5.23	27.07 ± 6.05
Lactate (mmol/L)	8.85 ± 3.92	8.79 ± 2.66	8.35 ± 4.38	10.43 ± 3.24	11.40 ± 2.61
Na+ (mEq/L)	145.53 ± 3.44	145.62 ± 4.15	143.67 ± 2.25	145.00 ± 4.29	144.67 ± 1.53
K+ (mEq/L)	6.13 ± 1.35	6.63 ± 1.29	6.57 ± 1.40	5.53 ± 0.81	9.53 ± 0.68

WBC: white blood cell, NEU: neutrophil, LYM: lymphocyte, MONO: monocyte, EOS: eosinophil, BASO: basophil, RBC: red blood cell, Hb: hemoglobin, MCV: mean corpuscular volume, ALB: albumin, pCO_2_: venous carbon dioxide partial pressure, HCO_3_^−^: bicarbonate, Na^+^: sodium, K^+^: potassium. ^†^ Kruskal–Wallis test was used for comparing two or more independent samples of different sample sizes. A significant result indicates that at least one sample stochastically dominates one other sample. All statistically significant indices were marked by a “cross shape”. Mann–Whitney test was used for comparing only two samples acquired from different times by double checking ^a^ *p* < 0.05 (before Asian sand dust (ASD) exposure vs. after 72 h) and ^b^ *p* < 0.05 (before ASD exposure vs. after 72 h).

**Table 2:** Distribution pattern of particles in lung tissue after exposure of artificial Asian sand dust.

Airway	Before Exposure of Artificial ASD		After Exposure of Artificial ASD							
			6 h		12 h		24 h		72 h	
	N = 16		N = 12		N = 9		N = 6		N = 3	
	Lt	Rt	Lt	Rt	Lt	Rt	Lt	Rt	Lt	Rt
Bronchus	0% (0/19)	0% (0/14)	40.0% (12/30)	46.4% (13/28)	42.4% (14/33)	33.3% (10/30)	20.8% (5/24)	25.6% (7/27)	20% (5/25)	19.4% (6/31)
Bronchioles	0% (0/37)	0% (0/37)	77.5% (62/80)	72.4% (55/76)	63.2% (36/57)	56.7% (34/60)	52.8% (28/53)	51.0% (25/49)	36.2% (21/58)	29.6% (16/54)
Alveolus	0% (0/120)	0% (0/120)	65.8% (158/240)	60.9% (140/230)	60.0% (96/160)	56.8% (84/148)	45.0% (81/180)	39.4% (71/180)	33.9% (81/180)	22.8% (41/180)

Lt: Left lung lobes, Rt: Right lung lobes.
